# A universal reference sample derived from clone vector for improved detection of differential gene expression

**DOI:** 10.1186/1471-2164-7-109

**Published:** 2006-05-05

**Authors:** Rishi L Khan, Gregory E Gonye, Guang Gao, James S Schwaber

**Affiliations:** 1 Daniel Baugh Institute for Functional Genomics/Computational Biology, Department of Pathology, Anatomy and Cell Biology, Thomas Jefferson University, Philadelphia, Pennsylvania 19107, USA; 2 Department of Electrical Engineering, University of Delaware, Newark, Delaware 19716, USA

## Abstract

**Background:**

Using microarrays by co-hybridizing two samples labeled with different dyes enables differential gene expression measurements and comparisons across slides while controlling for within-slide variability. Typically one dye produces weaker signal intensities than the other often causing signals to be undetectable. In addition, undetectable spots represent a large problem for two-color microarray designs and most arrays contain at least 40% undetectable spots even when labeled with reference samples such as Stratagene's Universal Reference RNAs™.

**Results:**

We introduce a novel universal reference sample that produces strong signal for all spots on the array, increasing the average fraction of detectable spots to 97%. Maximizing detectable spots on the reference image channel also decreases the variability of microarray data allowing for reliable detection of smaller differential gene expression changes. The reference sample is derived from sequence contained in the parental EST clone vector pT7T3D-Pac and is called vector RNA (vRNA). We show that vRNA can also be used for quality control of microarray printing and PCR product quality, detection of hybridization anomalies, and simplification of spot finding and segmentation tasks. This reference sample can be made inexpensively in large quantities as a renewable resource that is consistent across experiments.

**Conclusion:**

Results of this study show that vRNA provides a useful universal reference that yields high signal for almost all spots on a microarray, reduces variation and allows for comparisons between experiments and laboratories. Further, it can be used for quality control of microarray printing and PCR product quality, detection of hybridization anomalies, and simplification of spot finding and segmentation tasks. This type of reference allows for detection of small changes in differential expression while reference designs in general allow for large-scale multivariate experimental designs. vRNA in combination with reference designs enable systems biology microarray experiments of small physiologically relevant changes.

## Background

Microarray results enable systems biology only to the extent that they have (1) sensitivity/repeatability to detect low physiological-range regulatory events, (2) global detectability of spots to reveal broad system behavior, and (3) flexibility to support multivariate experimental designs. The present paper reports a new technical approach that improves microarray performance in all three of these areas. Due to variations in geometry, amount of DNA, hybridization efficiency, and background fluorescence at each spot, absolute fluorescence is not a reliable measure of mRNA abundance. However, if two samples are labeled with different dyes and co-hybridized to the same microarray, the ratio of their fluorescence intensities is a reliable measure of the differential RNA abundances between the samples. There are three typical designs used in two-color microarray experiments: dye swap, loop, and reference designs [1] and their advantages and disadvantages have been discussed in previous literature [2-4]. Loop and dye swap designs are useful for small studies (less than 5–10 samples) because they reduce variance and make full use of hybridization resources [2]. For larger studies, reference designs enable flexibility of (1) comparing all samples to each other through a single reference sample (2) expanding the design if more samples are needed (3) handling multivariate experimental designs such as time series or classification of multiple conditions [5,6]. Also, dye bias ((1) intensity dependant and (2) gene specific) is less of an issue in a reference design because (1) the dye incorporation effects cancel out in across array calculations in a manner similar to dye swap calculations and (2) gene-dye interactions are not an issue because the sample of interest is only measured on one dye. The reference design has been used successfully in a number of large microarray experiments [7-9].

An ideal reference RNA should (1) provide strong signal intensity to every probe on the microarray (2) be reliably reproducible in large batches (3) allow for comparison of datasets across laboratories and (4) mimic the hybridization characteristics of the biological sample it is compared against. Researchers typically prepare their own reference from pooled experimental samples or cell lines [7-10]. However these approaches are not easily reliably reproducible between labs and provide detectable signal for only 60–70% of the spots on a typical genomic-scale array. A reference sample derived from a mixture of cell lines is commercially produced by Stratagene called Universal Reference RNA™ (Stratagene, La Jolla, CA) and is a commonly used reference RNA but still only yields detectable signal for 60–70% of the spots on most microarrays [11]. Other groups have used genomic DNA [12,13], a mixture of clones spotted on the arrays [14,15], and a short oligomer that is complementary to every spot on the microarray [16]. However, a mixture of clones is specific to array design and thus is generally not usable between laboratories and short oligos do not have the same hybridization characteristics as longer RNA molecules in biological samples [17]. While a genomic DNA reference does mimic cDNA hybridization characteristics, it represents a heterogeneous sequence distribution and requires a different reference for each species. None of these three methods are widely used and publicly available data is not widely available. Therefore we focus our comparison with Stratagene Universal Reference.

In the present approach, we describe a universal RNA reference that possesses features 1–4 discussed above. This universal reference RNA contains a sequence from the vector that is common to all of the cDNA spotted on the slides but does not contain any specific gene sequence. We call this reference sample "vector RNA" (vRNA) and its use in a reference design "vector reference design". Using vRNA provides strong signal for every spot on a microarray. This allows for within-slide quality control of printing errors, large hybridization inefficiencies and within-batch quality control of insufficient concentrations of spotted DNA on a per spot basis. Finally, use of vRNA as a reference assists in spot finding because every spot has a detectable signal.

## Results

### Development of vRNA sample

**Figure 1:**
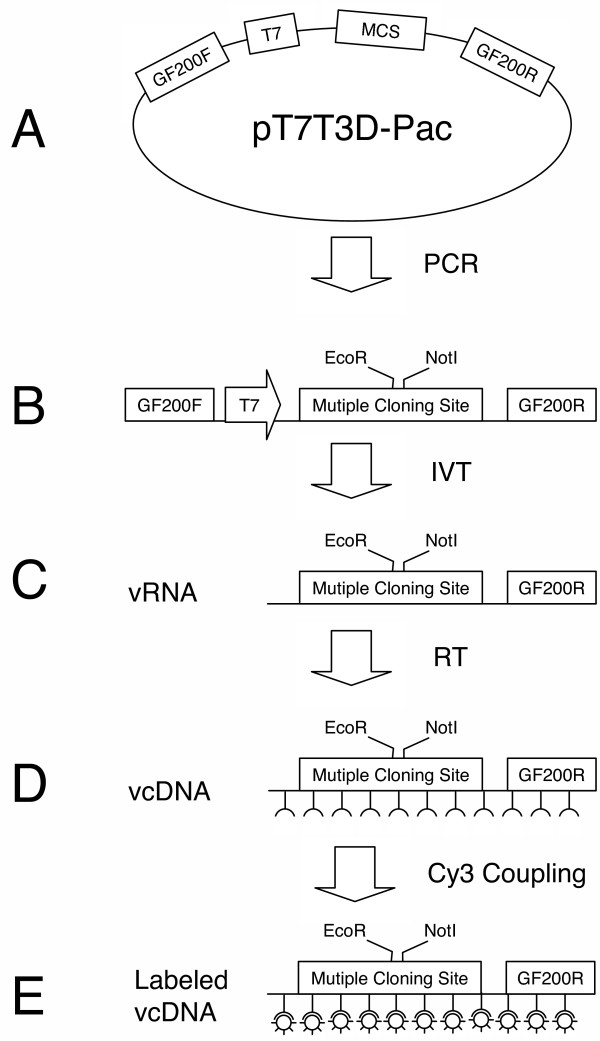
Schematic of vRNA production. (a) The parental EST clone vector (pT7T3D-pac) is used as a template for PCR with GF200 primers. (b) The PCR product is further amplified and converted to aRNA via an in vitro transcription reaction. (c) The aRNA is reverse transcribed and incorporated with amino allyl dUTP creating vcDNA. (d) The vcDNA is coupled with Cy3 dye. (e) The product of eight labeling reactions are combined (20 uL) and diluted to 125 uL.

We obtain a homogeneous RNA sample from the parental EST clone vector as described in Methods and outlined in Figure 1. This reference RNA (vRNA) contains a 220 base pair sequence from the vector that is common to all of the cDNA spotted on the slides, but does not contain any specific gene sequence. Oligomers larger than 60 bases have similar hybridization characteristics as biological RNA [17]. vRNA was tested on 40 microarrays and was found to reproducibly yield strong signal for almost every spot on the array.

### Detectability

We call a spot detectable if it has a signal-to-noise ratio (SNR) larger than three (see Methods) and define percent detectability as the fraction of all spots on an array that are detectable. For comparison to other results [11], we also calculate percent spots with a signal to background ratio (SBR) greater than two. We studied cDNA and oligonucleotide microarray datasets in the Gene Expression Omnibus (GEO) [18] that used StrataGene's Universal Reference RNA™. A search on GEO of "Stratagene Universal Reference" yields 44 experiments (as of October 31, 2005). The subset of these that contain background signal standard deviation information were analyzed for signal detectability using SNR and signal-to-background ratio (SBR). The results are presented in Table 1 (rows 1 through 20). On average in these datasets, only 60% of the genes had detectable spots (range was 14% to 87% detectable spots, see Table 1). vRNA empirically is calibrated such that almost all spots are detectable (see Methods). Table 1 shows that the vRNA dataset has 97% detectable spots on the vRNA channel (Cyanine-3; Cy3). Typically additive background noise corrupts the measurements of one dye more than the other affecting the detectability of the signal [19,20]. Therefore, using vRNA with the weaker dye can greatly increase the overall detectability of spots.

### Calibration of vRNA sample

**Figure 2:**
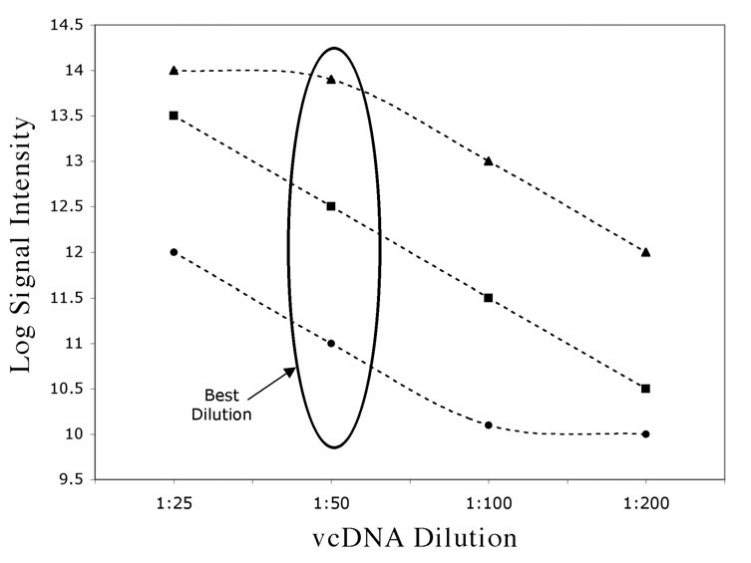
Signal intensity (log_2_) of high (▲), medium (■) and low (●) mRNA abundances vs dilution. 1:25 dilution saturates high abundance RNA samples whereas 1:200 dilution results in undetectable signal for low abundance RNA. High, medium and low mRNA abundances. 1:50 dilution produces detectable but not saturated signal for the most spots.

We performed a titration experiment using concentrations 1:25, 1:50, 1:100, and 1:200 of vcDNA prepared (as described in Methods). Ideally, signal intensities for all spots should decrease 50% with each dilution. A ratio of less than two suggests either saturation or loss of detectability, and thus the best dilution is the one that yields the highest signal intensity in the linear regime of the dilution curve. Figure 2 illustrates the intensities of low, medium, and high intensity spots over different vcDNA dilutions. The dilution that was the best for the majority of the spots was 1:50.

### Narrow dynamic range of vRNA signal intensity

**Figure 3:**
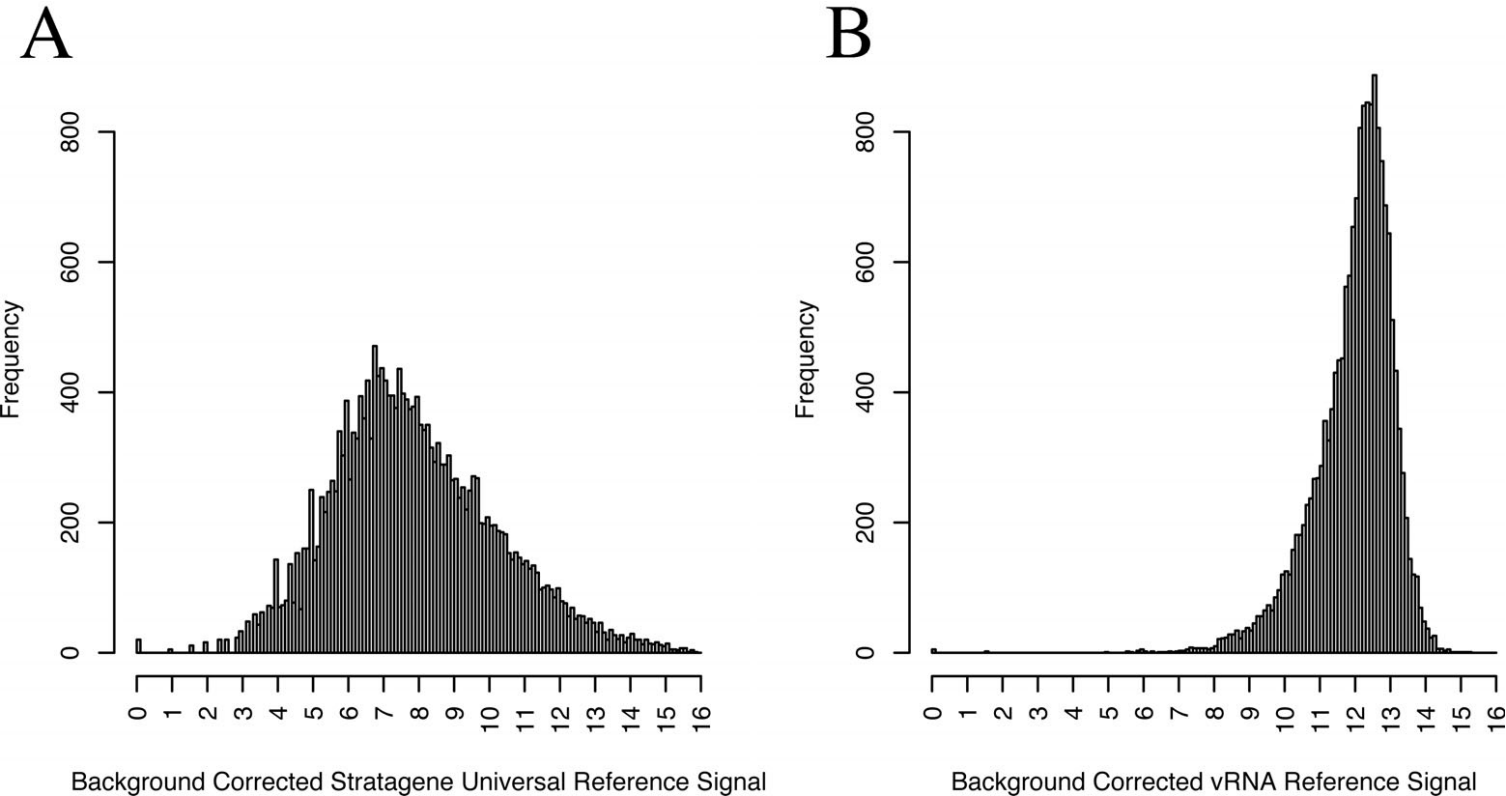
Histograms of background corrected spot intensities of reference samples. (a) Stratagene Universal Reference RNA™ from dataset GSE1706 (b) vRNA. Note that the range of background corrected spot intensities in a biologically derived reference (Stratagene Universal Reference RNA™) cover 11 orders of magnitude and the middle 50% of the spot intensities cover 5 orders of magnitude whereas background corrected spot intensities using vRNA covers 7 orders of magnitude and middle 50% of the spot intensities cover 2 orders of magnitude.

We compared the dynamic range of datasets using Stratagene Universal Reference RNA™ as reference to our vector reference design with respect to the metric of detectability without saturation. The spot intensities obtained from GEO datasets (Table 1) using Stratagene's Universal Reference RNA™ ranges from undetectable to saturation. However, spot intensities of vRNA are more consistent, never saturated, and undetectable only when the concentration of spotted PCR product is insufficient. Figure 3 shows histograms of background corrected spot intensities of microarray experiments representative of using Stratagene's Universal Reference RNA™ (from dataset GSE1706; this dataset is typical, other datasets shown in Supplementary Figure 1) and vRNA. We also expect the dynamic range of vRNA signal intensity to be smaller than that of genomic DNA reference because vRNA is homogenous. vRNA allows for more flexibility in calibration than a biologically derived reference because the vcDNA is homogeneous and the resulting signal intensities have a smaller dynamic range.

### Comparison of reference designs using biologically derived reference and vRNA

Stratagene's Universal Reference RNA™ (Stratagene, La Jolla, CA) is a commonly used biologically derived reference for use in reference designs [5]. We studied cDNA and oligonucleotide microarray datasets in GEO that used StrataGene's Universal Reference RNA™. All 20 datasets in Table 1 showed a statistically significant difference in detectability between dyes. Most groups show improved Cy5 signal detectability over Cy3 (range between laboratories: 2%–33%, mean difference: 6%, p < 0.0001). In typical cases where detectability is significantly different between the dyes, it is clear that vRNA can improve the detectability of the weaker channel thus boosting the fraction of genes that produce detectable signal. We have calculated detectability measurements for an arbitrary sample of GEO experiments that use pooled biological samples as a reference and obtained similar results (data not shown).

### Value of the vector reference design with respect to quality control issues

vRNA can be used as a quality control sample. By hybridizing labeled vcDNA with saturating concentrations in vast excess of the DNA spotted on the slide, all of the spots containing clones should show spot intensities proportional to the amount of DNA competent for hybridization in the spot. In the vRNA design those spots that do not have signal intensities much higher than background (by simple manual visualization, or by statistical measures such as SNR) represent clones that have insufficient concentrations of PCR product possibly due to failed or inefficient PCR reactions, no bacterial growth, or robotic printing errors. Rouse et al. describes a reagent derived from synthesized oligonucleotides similar to vRNA in order to quantitate the amount of cDNA printed in each array element and subsequently determine the molar stoichiometry of the target cDNA bound to the probe molecules available for hybridization [21].

In a study of ethanol adaptation using vRNA [22], the median fraction of detectable spots using vRNA was 86% (Table 1; GSE2718). About 10% of the spots contained insufficient concentrations of PCR product and were easily distinguishable from other spots using a SNR threshold of 3 (Supplementary Figure 2). After rearraying our slides and adding higher concentration PCR product to spots with low signal intensity from the vRNA sample, the median Cy5 detectability was increased to 95% and the Cy3 detectability was increased to 97% (Table 1 – vRNA).

**Figure 4:**
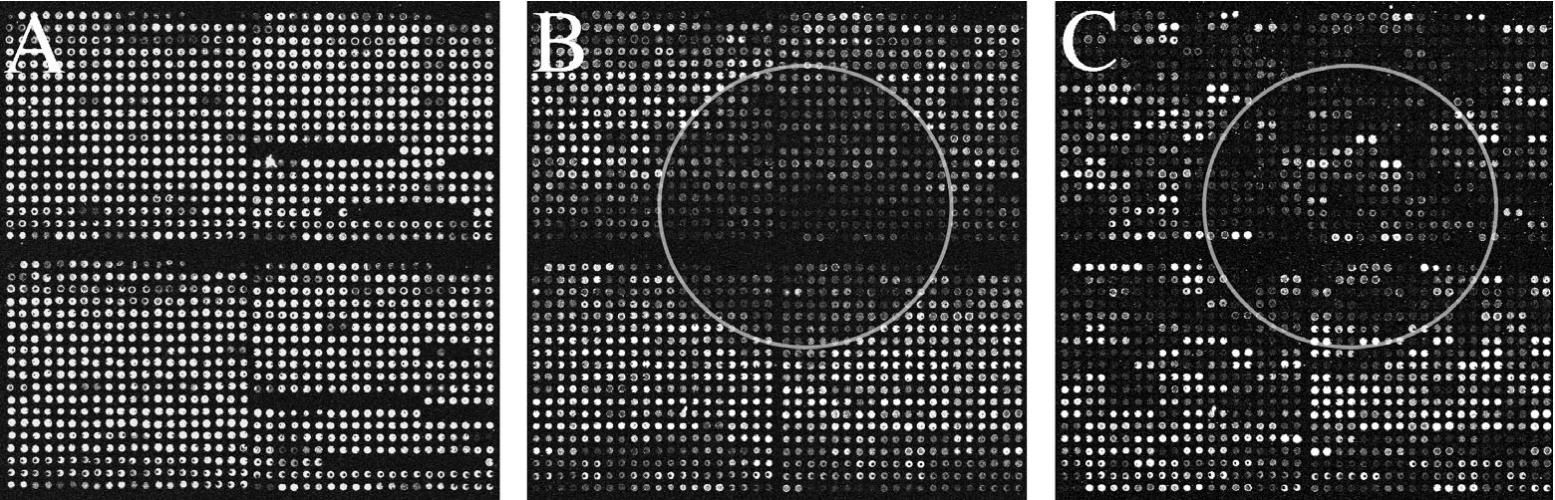
Microarray images showing technical errors detected by vRNA. (a) The two subarrays on the right show two rows each that were not printed. Additionally there are several weaker spots that indicate less PCR product spotted in those locations. (b) Image of vRNA sample channel showing a large area of low signal intensities (a portion enclosed by the white circle) where labeled sample did not hybridize well to the slide, possibly due to an air bubble. (c) Image of the biological sample from the same microarray. It is not easy to see a hybridization problem on this channel because of the large variation of signal intensities.

vRNA can identify printing errors. While insufficient PCR product from printing plates yield undetectable spots on every slide, some slides contain missing spots specific to that slide due to a robotic printing error where the print tip did not touch that microarray and deposit cDNA. vRNA provides an in-slide control for all printing errors. The data from spots not printed should not be considered in subsequent microarray analysis. Figure 4a shows an example of spots not printed as detected by the vRNA sample channel.

Finally, the vRNA can help detect hybridization inconsistencies by showing non-uniformities in the image. Figure 4b illustrates an artifact caused by an air bubble as seen on the vRNA sample channel. This anomaly is not visually obvious in the image of the biological sample channel (Figure 4c) and, by inference, in the image of a biologically derived reference sample (Stratagene or pooled biological samples). The overall uniformity of signal intensities resulting from using vRNA makes it possible for researchers to quickly identify and flag these problem areas of the microarray. As with other reference designs, the reference channel may help to normalize these anomalies from slide to slide, but if the anomaly is visibly clear, we suggest flagging those spots as bad spots and removing these features from downstream analyses.

### vRNA and spot finding

Using vRNA yields a bright signal for each spot (barring issues described in the quality control section) and therefore makes spot finding much simpler. By thresholding the image by some large pixel intensity that is well above the background intensity level but within the range of the vRNA signal (around 2000 in our experience), all contiguous objects with over 50 pixels represent a spot or a bright artifact (Figure 4). The spots are uniformly distributed with a grid pattern (within tolerance of small printing variability) and because over 90% of the spots should be bright (even considering array quality control issues), detecting the subarrays and the positions of each spot is straightforward. Since the channel representing the vRNA sample contains the same physical layout as the channel representing the biological sample, the same spot location and segmentation data derived from the reference channel can be applied to the biological sample channel.

## Discussion

Based on our analysis of publicly available datasets from GEO, the vector reference design improves on previous approaches to reference design. The vRNA results show improved detectability compared to traditional references such as the Stratagene Universal Reference RNA™ or pooled biological samples. We measured detectability as a function of signal-to-noise ratio (SNR) and signal-to-background ratio (SBR). The second measure (SBR) is not as statistically rigorous and can be biased by adjusting the mean background signal up or down, but using it allows one to also consider datasets which do not include background intensity standard deviations, which is typical of most datasets publicly available in GEO (as of October 31, 2005). One dataset (GSE1818; see table 1) was excluded due to atypical background issues. For all cases evaluated, vRNA provides higher detectability than the other references used.

We showed how vRNA could be used for spot finding and for quality control of PCR product generation, batch printing, array manufacture, protocol evaluation, and individual hybridizations. The vRNA sample channel image can be visually inspected to ensure that there are no artifacts caused by technical problems. Spots with low signal detectability should be flagged as bad spots and excluded from downstream analyses. Commercial references and pooled sample references do not lend themselves to these quality control features because they contain many genes with low expression and thus produce many spots that are not detectable. Therefore anomalies that are apparent using vRNA as a reference are hidden when using other reference samples.

Reference samples yielding detectable but not saturating signals for all spots have been previously reported. Several groups created references from the clones used to make their microarrays. The first approach, outlined by Dudley et al [16], uses a 25 mer oligomer that matches a small portion of the parental EST clone vector that is contained in every PCR product printed on the microarray. Specifically, it matched the PCR primer used to make PCR product from the clones. However, the melting point of hybridized oligomers increases with length up to about 60 bases [17]. Therefore, the characteristics of hybridization to the spotted cDNA are different between a 25 base oligomer and a typical cDNA (~100–1500 bases). Ideally, a reference should have identical hybridization characteristics to biological cDNA to control for hybridization variability.

A second method of creating a universal reference from the characteristics of the clone is described by Sterreburg et al. [14]. Briefly, they suggest pooling all clones together in a single tube, performing a PCR reaction to create in vitro transcription template for all of the cDNA inserts, *in vitro *transcription of the PCR product, DNase treatment, reverse transcription, and labeling. This produces a reference sample representing all of the sequences on the array. Each sequence in the reference sample still contains the flanking regions of the parental EST clone vector. Gorreta et al. [15] simplifies this process by simply labeling the PCR product. Both of these reference samples yield over 90% detectability of all spots on the array whereas the Stratagene Universal Reference RNA™ sample produces 50% detectability [15]. However, reference sample produced by both of these methods cannot be used to compare datasets across laboratories.

We developed a new universal reference combining and extending the advantages of Sterrenburg et al. [14], Gorreta et al. [15], and Dudley et al. [16] show that only the sequence common to all of the PCR products (i.e. the sequence in the parental EST clone vector between the PCR primers) is needed to provide a strong detectable signal and reliable measure of hybridization and printing variability. We have developed a method to make such a reagent that is quick, cheap, repeatable, and effective. Rouse et al. uses a similar reagent, in large molar excess to probe molecules, in a method which attempts to determine a stoichiometric molar ratio between hybridized cDNA and available probe molecules spotted on the array [21]. Our universal reference increases the detectability of spots on an array to an average of 97%. This is comparable to their results [14,15] while requiring significantly less effort and money and providing a homogeneous RNA sample. Finally, although the concept of using a vRNA reference is universal, one must ensure the appropriate parental sequence is used. A commercially produced vRNA sample would allow for comparison of datasets across laboratories using cDNA microarrays that use the same parental vector. pT7T3D-Pac vectors are commonly used in clone libraries. 50% of all rat clones, 25% of all mouse clones, and 25% of all human clones use the pT7T3D-Pac vector.

We have presented a method for creating a vRNA sample using clones containing the pT7T3D-Pac vector. vRNA can be made for clones that use other vectors as well. The T7 RNA amplification step requires a T7 promoter, which is present in many vectors. Alternatively, PCR can be used to replace the in vitro transcription (IVT) step. Multiple vectors (e.g. pT7T3D-Pac, pSPORT1, and pCMV-SPORT6) can be mixed together to create a universal reference that can be used on 75% of all rat, mouse, and human clones.

Vector reference designs can only be used in microarrays that are spotted with cDNA that have common sequence, such as PCR product generated from clones. Affymetrix arrays typically use one dye and are not amenable to reference designs because they contain short (16–25 mer) gene specific sequences with no common sequence on all array features. Spotted oligonucleotide arrays such as Operon (Operon Biotechnologies, Huntsville, AL) or Compugen/Sigma (Compugen USA, San Jose, CA; Sigma Co., St. Louis, MO) do not contain a common sequence in each spot. In the future, such a 60 base sequence could be manufactured. Currently 110 bases can be efficiently synthesized (Sigma). Therefore, a small improvement in oligonucleotide synthesizing techniques could yield 140 base oligonucleotides that contain 70 bases of a gene of interest and 70 bases of a common sequence. *In situ *oligonucleotide arrays such as Agilent arrays could, in principle, be designed in a similar manner. However, current commercially available *in situ *oligonucleotide synthesis arrays are limited to 60 base oligomers (Agilent Technologies, Palo Alto, CA).

vRNA reference designs increase reproducibility across experiments because the reference signal is detectable for all spots on the array. This increased reproducibility of data across arrays increases the sensitivity of the differential gene expression measurements and enables system-wide detection of small, physiologically relevant changes in gene expression.

## Conclusion

Results of this study show that a reference sample (vRNA) derived from the parental EST clone vector of all clones printed on an array provides a useful universal reference that can be used for quality control of microarray printing and PCR product quality, detection of hybridization anomalies, and simplification of spot finding and segmentation tasks. vRNA can be made inexpensively in large quantities as a renewable resource that is consistent across experiments. This type of reference allows for detection of small changes in differential expression while reference designs in general allow for large-scale multivariate experimental designs. vRNA in combination with reference designs enable systems biology microarray experiments of small physiologically relevant changes.

## Methods

### Universal vector reference generation

The EST clones used to manufacture the cDNA arrays described herein all used the pT7T3D-Pac vector (generously provided by the BMAP group at University of Iowa). Therefore, they all contained the same sequence between the viral promoters (T3 and T7) and the multicloning site between the Notl and EcoRI. PCR of an empty vector using GF200 primers (5'-CTGCAAGGCGATTAAGTTGGGTAAC-3' and 5'-GTGAGCGG-ATAACAATTTCACACAGGAAACAGC-3') yields a template for T7 based RNA amplification (MessageAmp™ T7 Linear Amplification Kit, Ambion, Austin, TX) to produce "vector RNA" (vRNA). vRNA was reverse transcribed (detailed further below) with dNTPs, amino allyl-dUTP and random nanomers and coupled with the monoreactive succinimide ester derivative of a Cy3 or Cy5 dye to create labeled vector derived cDNA (vcDNA).

### RNA sources

RNA sources used to generate datasets obtained from GEO are described in their respective papers (Table 1). RNA for the in house dye swap and the reference data were collected from male Sprague-Dawley rats from Charles River Laboratories (Wilmington, MA) housed at the Animal Core Facility of the Thomas Jefferson University. The animals were sacrificed by rapid decapitation and the nucleus tractus solitarius (NTS) was isolated by microdissection. Total RNA was extracted using Qiagen's RNeasy mini kit (Qiagen, Valencia, CA), yielding 200–900 ng of total RNA. RNA quality was assessed using a RT-PCR protocol for high and low copy number genes (β-actin and tyrosine hydroxylase respectively). Tyrosine hydroxylase was selected because it is specific to the NTS at the slice level and punch region, confirming that the punches contained the NTS.

### Microarray manufacture

Microarrays were fabricated using a rat clone set (GF200; ResGen Huntsville, AL) for cDNA microarrays consisting of approximately 1900 sequence-verified non-redundant cDNA clones (as of Unigene build 78) and an additional 6900 clones from Invitrogen (Invitrogen, Carlsbad, CA) for a total of 8800 clones. cDNA probes from EST clones were prepared from freshly grown overnight bacterial cultures by PCR amplification using GF200 primers (Invitrogen). PCR products were purified and verified by agarose gel electrophoresis, and the yield was determined spectrophotometrically (NanoDrop Wilmington, DE). cDNAs were mixed with equal volume of DMSO (10–70 ng/μl) and printed onto FMB cDNA slides (Full Moon Biosystems, Sunnyvale, CA) using a MicroGrid II arrayer (Genomic Solutions, Ann Arbor, MI). Microarrays were air dried for 30 min and cross-linked by UV irradiation. We printed arrays of 18,240 spots representing 8832 clones and 288 internal controls in adjacent duplicate spots.

### RNA amplification and labeling

Total RNA (70–400 ng) was amplified using two rounds of the antisense RNA (aRNA) technique [23], yielding on average 180 μg aRNA (MessageAmp, Ambion, Austin, TX). aRNA (1.125 μg) was reverse transcribed (Superscript II, Invitrogen) using random primers to generate single stranded amino-allyl derivatized cDNA, which was coupled with Cy dyes (Cy3 or Cy5) to produce fluorescently labeled cDNA.

### Hybridization

Microarrays were prehybridized in 1% bovine serum albumin, 5× SSC, 0.1% SDS for 45 min at 42°C, washed in H_2_O and dried by centrifugation. Cy3 labeled vDNA and Cy5 labeled cDNA samples were mixed with 50 μl of Dig Easy Hybridization buffer (Roche, Indianapolis, IN) containing 25 μg each of yeast tRNA and calf thymus DNA and applied to the microarrays for hybridization at 37°C for 16 hours in a hybridization chamber (Corning, Corning, NY) in the dark with gentle agitation. Slides were washed for 10 minutes at 50°C in 1× SSC and 0.1% SDS in shaking incubator, followed by a 1 minute wash in 1× SSC, three 1 minute washes in 0.1× SSC, and one rinse in H_2_O, at room temperature. Slides were dried by centrifugation and scanned with a ScanArray 5000 XL (PerkinElmer, Wellesley, MA). Image analysis was performed using ScanArray Express v2.2 software.

### Dilution calibration

In 8 tubes, 8 aliquots of 2 μg of vRNA sample was converted to Cy3 labeled vcDNA as described above. The labeled reference was pooled (20 μL final volume). Dilutions of 1:25, 1:50, 1:100, and 1:200 of this pooled sample were combined with water (2.5 μL final volume per dilution), hybridized to four separate arrays, scanned, and quantitated.

### Data analysis

#### Spot quantitation from images

We quantitated the scanned images with ScanArray Express 2.0 using the adaptive thresholding quantitation algorithm to generate values for median signal intensity, median background intensity, and background intensity standard deviation for each spot on the array. In this analysis, no spots were flagged, but the array data was visually inspected and arrays with atypical signal were discarded and redone. For each dataset used from GEO, the spot quantitation and software and algorithms are discussed in each dataset's corresponding publication (Table 1).

### Detectability metrics

We model measured signal intensity (log-normalized) as the underlying real signal corrupted by white noise:

measured signal intensity = true signal - N(μ_b _- σ_b_)

Where μ_b _is the mean background intensity and σ_b _is the standard deviation of the background intensity. We wish to calculate signal detectability. There are three ways this is done in the literature. (1) From signal processing [4], the detectability of a signal can be determined by the signal-to-noise ratio (SNR)which is:

mean signal intensity−mean background intensitystandard deviation of background intensity
 MathType@MTEF@5@5@+=feaafiart1ev1aaatCvAUfKttLearuWrP9MDH5MBPbIqV92AaeXatLxBI9gBaebbnrfifHhDYfgasaacH8akY=wiFfYdH8Gipec8Eeeu0xXdbba9frFj0=OqFfea0dXdd9vqai=hGuQ8kuc9pgc9s8qqaq=dirpe0xb9q8qiLsFr0=vr0=vr0dc8meaabaqaciaacaGaaeqabaqabeGadaaakeaadaWcaaqaaiabb2gaTjabbwgaLjabbggaHjabb6gaUjabbccaGiabbohaZjabbMgaPjabbEgaNjabb6gaUjabbggaHjabbYgaSjabbccaGiabbMgaPjabb6gaUjabbsha0jabbwgaLjabb6gaUjabbohaZjabbMgaPjabbsha0jabbMha5jabgkHiTiabb2gaTjabbwgaLjabbggaHjabb6gaUjabbccaGiabbkgaIjabbggaHjabbogaJjabbUgaRjabbEgaNjabbkhaYjabb+gaVjabbwha1jabb6gaUjabbsgaKjabbccaGiabbMgaPjabb6gaUjabbsha0jabbwgaLjabb6gaUjabbohaZjabbMgaPjabbsha0jabbMha5bqaaiabbohaZjabbsha0jabbggaHjabb6gaUjabbsgaKjabbggaHjabbkhaYjabbsgaKjabbccaGiabbsgaKjabbwgaLjabbAha2jabbMgaPjabbggaHjabbsha0jabbMgaPjabb+gaVjabb6gaUjabbccaGiabb+gaVjabbAgaMjabbccaGiabbkgaIjabbggaHjabbogaJjabbUgaRjabbEgaNjabbkhaYjabb+gaVjabbwha1jabb6gaUjabbsgaKjabbccaGiabbMgaPjabb6gaUjabbsha0jabbwgaLjabb6gaUjabbohaZjabbMgaPjabbsha0jabbMha5baaaaa@A14D@

(2) Another method uses the mean and standard deviations of the mean signal intensities of all negative control spots as a surrogate for mean and standard deviation of background intensity of each spot [15]. This method allows for the possibility of some spot autofluorescence but does not account for local background effects. (3) Stratagene and others [11] use the ratio mean signal intensity to mean background intensity (signal-to-background ratio or SBR). This measure can be biased by adjusting the offset on the photomultiplier tube, effectively adding or subtracting a constant to both the signal and background. However, it may be the only option in analyzing datasets where standard deviation of background signal is not given.

Using SNR as defined by [4] as a metric, we consider a spot detectable if the SNR > 3 (following [4] and signal processing literature). Using SBR as a metric, we consider a spot detectable if the SBR > 2, which produces similar results as SNR > 3 and is used by [11] and Stratagene). Method (2) is difficult to use on a wide variety of public microarray datasets because it is often difficult to interpret the meaning of the controls. Some laboratories call a location that was not spotted as empty while others call a location with a spot of DMSO as empty. We only calculate the detectability of spots representing genes of interest and exclude control spots from this analysis.

## Authors' contributions

RK conceived of using vRNA as a reference sample, carried out the molecular biology experiments, created the experimental design, performed the microarray analysis and drafted the manuscript. GEG developed the initial protocol for vRNA production, established vRNA's use as a quality control, and helped draft the manuscript. GG mentored RK in statistical measures of detectability and directed the analysis and comparison to other datasets. JS directed the project, participated in its design and coordination, and helped draft the manuscript. All authors read and approved the final manuscript.

## Conflicts of interest

The author(s) declare that they have no competing interests.

## Figures and Tables

**Table 1:** Signal to noise ratio (SNR) and signal to background ratio (SBR) detectability metrics microarray datasets. The first 20 microarray datasets use Stratagene Universal Reference RNA™ as a reference. The bottom 2 microarray datasets are datasets generated by our laboratory

		SNR > 3			SBR > 2			
			
Experiment	Platform	Cy3	Cy5	Both	Cy3	Cy5	Both	Pubmed ID
GSE332	GPL260	89%	88%	87%	68%	67%	64%	
GSE333	GPL260	89%	80%	80%	66%	49%	47%	
GSE432	GPL285	55%	24%	24%	42%	25%	24%	
GSE1041	GPL981	65%	48%	47%	75%	68%	66%	
GSE1138	GPL977	58%	48%	42%	82%	72%	69%	15156144
GSE1319	GPL1197	91%	83%	83%	86%	88%	84%	15328174
GSE1486	GPL1225	33%	27%	19%	18%	37%	16%	
GSE1533	GPL1299	20%	19%	14%	25%	34%	21%	
GSE1645	GPL1384	54%	51%	46%	63%	63%	58%	15718295
GSE1705	GPL1390	96%	86%	85%	91%	87%	87%	
GSE1705	GPL885	87%	59%	58%	80%	71%	70%	
GSE1705	GPL887	71%	61%	59%	67%	79%	65%	
GSE1706	GPL1390	88%	75%	74%	81%	83%	77%	15679094
GSE1706	GPL885	88%	78%	76%	75%	79%	72%	15679094
GSE1706	GPL887	76%	54%	53%	71%	56%	56%	15679094
GSE1818	GPL885	100%	100%	100%	100%	97%	97%	15994931
GSE2134	GPL1786	86%	89%	83%	89%	89%	87%	
GSE2900	GPL2614	72%	88%	70%	80%	91%	80%	15838786
GSE3023	GPL2695	78%	86%	74%	85%	94%	85%	16168081
GSE3023	GPL2696	82%	86%	81%	88%	89%	87%	16168081

GSE2718	GPL2000	86%	74%	71%	82%	73%	67%	16189278
vRNA	in-house	97%	95%	93%	97%	90%	87%	
